# The Influence of Laser Ablation Parameters on the Holes Structure of Laser Manufactured Graphene Paper Microsieves

**DOI:** 10.3390/ma13071568

**Published:** 2020-03-28

**Authors:** Barbara Nasiłowska, Zdzisław Bogdanowicz, Antoni Sarzyński, Wojciech Skrzeczanowski, Małgorzata Djas, Bartosz Bartosewicz, Bartłomiej J. Jankiewicz, Ludwika Lipińska, Zygmunt Mierczyk

**Affiliations:** 1 Institute of Optoelectronics, Military University of Technology, gen. S. Kaliskiego 2, 00-908 Warsaw, Poland; a.sarzynski9@upcpoczta.pl (A.S.); wojciech.skrzeczanowski@wat.edu.pl (W.S.); bartosz.bartosewicz@wat.edu.pl (B.B.); bartlomiej.jankiewicz@wat.edu.pl (B.J.J.); zygmunt.mierczyk@wat.edu.pl (Z.M.); 2 Faculty of Mechanical Engineering, Military University of Technology, gen. S. Kaliskiego 2, 00-908 Warsaw, Poland; zdzislaw.bogdanowicz@wat.edu.pl; 3 Łukasiewicz Research Network–Institute of Electronic Materials Technology, Department of Chemical Synthesis and Flake Graphene; Wólczyńska 133, 01-919 Warsaw, Poland; malgorzata.djas@itme.edu.pl (M.D.); ludwika.lipinska@itme.edu.pl (L.L.)

**Keywords:** graphene paper, graphene paper microsieves, picosecond laser, laser ablation, laser manufacturing

## Abstract

The graphene paper microsieves can be applied in the filtration of biological fluids or separation of solid particles from exploitation fluids. To produce graphene paper microsieves for specific applications, good control over fabrication should be achieved. In this study, a laser ablation method using a picosecond laser was applied to fabricate graphene paper microsieves. Holes in the microsieves were drilled using pulsed laser radiation with a pulse energy from 5 to 100 µJ, a duration of 60 ps, a wavelength of 355 nm, and a repetition rate of 1 kHz. The impact method was applied using 10 to 100 pulses to drill one hole. To produce holes of a proper diameter which could separate biological particles of a certain size (≥10 µm), optimum parameters of graphene paper laser ablation were defined using the MATLAB software taking into account laser pulse energy, repetition rate, and a desired hole diameter. A series of structural tests were carried out to determine the quality of an edge and a hole shape. Experimental results and Laguerre–Gauss calculations in MATLAB were then compared to perform the analysis of the distribution of diffraction fringes. Optimum experimental parameters were determined for which good susceptibility of the graphene paper to laser processing was observed.

## 1. Introduction

Graphene is a 2D crystal composed of a single layer of carbon atoms arranged in a hexagonal mesh. The development of efficient fabrication methods and the ability to determine its functional characteristics have resulted in an increasing interest in the application of graphene and its derivatives in many areas of technology and medicine [1,2,3,4,5]. Graphene paper shows, for example, a great promise for electrical energy storage [6,7,8,9]. Through the design of hierarchical architectures and interlayer interaction, multifunctional macroscopic graphene oxide papers also tend to achieve outstanding mechanical properties [10,11].

The available literature shows published studies in which a laser ablation process was carried out on the graphene deposited mainly by the chemical vapor deposition (CVD) method on various substrates [12,13,14], i.e., glass [15,16] and silicon [14,17,18,19]. In these papers the authors have shown, among other things, changes of graphene structure in the area of laser beam interaction. This is connected with partial melting of the substrate and graphene sublimation. Raman spectroscopy measurements were performed to identify transgression of the graphene layer [15,16,17]. This research indicates that in the area of laser beam interaction a disturbance of continuity and even breakage of the graphene shell occurs quite often. This is mainly connected with applied ablation parameters. During analysis of the results of the graphene laser ablation, it was observed that an enhancement of laser radiation damages the graphene surface in the heat outflow zone, while in the area of laser beam interaction the graphene surface is totally broken [14,15,16,17,18]. The importance of laser ablation parameters has been shown in studies on graphene patterning by nanosecond laser ablation [12]. When monolayer graphene samples supported on a Si/SiO_2_ substrate were patterned using 532 nm laser irradiation under a fluence of 356 mJ·cm^−2^, only the selective ablation of graphene was observed. However, when the fluence was increased to above 1030 mJ·cm^−2^, damage to substrate was observed instead of graphene ablation [12].

In the case of graphene paper composed of many layers of graphene and not deposited on the substrate, the substrate does not disturb its structure during laser ablation. However, the applied laser ablation parameters have influence on the shape and topography of the area of laser beam interaction. This is a very important factor when particular sizes and shapes of the holes are required for specific applications. Such a situation occurs, for instance, in the case of the microsieves made of graphene paper. During the production of microsieves from graphene paper, which can be applied in the filtration of biological fluids as well as separation of solid particles from exploitation fluids, selection of parameters for laser processing is an important issue [3,4,20]. Attempts to use the microsieves produced with the use of a laser ablation method showed that they allow for separation of those circulating tumor cells whose diameter does not exceed 10 µm. The hole diameter assumed in isolating biological particles should be then 6 to 10 µm [4]. Another important issue is the spacing of diffraction fringes. It is particularly essential since control of their spacing allows us to obtain a minimum distance between the holes without introducing structural changes so that the heat flow zone is not able to affect the adjacent hole.

In the literature, there are several reports on laser-assisted synthesis, reduction, and micro-patterning of graphene or graphene oxide [12,13], including fabrication of graphene oxide ultrathin flat lenses using direct laser writing with a femtosecond laser [21]. However, to the best of our knowledge, there are no studies in which the influence of laser ablation parameters on the hole structure of laser manufactured graphene paper microsieves has been investigated. In this paper, we describe the results of our studies on the theoretical and experimental determination of the graphene paper laser ablation parameters allowing the fabrication of microsieves with controlled diameters of the holes. Theoretical modeling in the MATLAB software has been used to model the laser ablation parameters. The modeling results have been then compared to results obtained experimentally. Results presented in the paper extend knowledge in the field of graphene paper modifications by a precise laser ablation.

## 2. Materials and Methods

### 2.1. Materials

The studies were conducted on graphene paper of the thickness in a range of 80–100 μm produced at the Department of Chemical Synthesis and Flake Graphene, Łukasiewicz Research Network—Institute of Electronic Materials Technology in Poland, according to patented technology [22]. A graphene oxide (GO) dense water suspension liquid, degassed and concentrated over 10 g/L, was used to produce graphene paper. The liquid GO was prepared by a modified Hummers’ process [23]. The process of obtaining the paper was performed using a twin-roll press (homemade tool, Łukasiewicz Research Network—Institute of Electronic Materials Technology, Warsaw, Poland). The graphene oxide paste was injected between the sheets of a suitable filtering fabric and then pressed. After this process, the sheets containing the GO paste and located between them were dried in controlled conditions at a temperature not exceeding 45 °C. Laser-Induced Breakdown Spectroscopy (LIBS, LLA Instruments GmbH, Berlin, Germany), Raman Spectroscopy (Renishaw plc., Wotton-under-Edge, UK), and Diffuse Reflectance Infrared Fourier Transform Spectroscopy (Spectrum GX FTIR Spectrometer, PerkinElmer Inc., Waltham, MA, USA) were used to determine chemical purity and composition of the fabricated graphene paper. The results of graphene paper characterization are provided in Supporting Information in Figures S1–S3.

### 2.2. Characterization of Graphene Paper by Scanning Electron Microscopy

The morphology of graphene paper before and after laser ablation was investigated by scanning electron microscopy (SEM) using a Quanta 250 FEG SEM, FEI, Hillsboro, OR, USA. SEM images were acquired using a backscattered detector (ETD-BSE, FEI, Hillsboro, OR, USA) with an accelerating voltage of 10 kV. The analysis of the SEM images allowed us to determine the kind and extent of damage arising as a result of laser beam interaction with graphene paper and optimization of size and shape of microholes generated during ablation.

### 2.3. Laser Ablation

**Figure 1:**
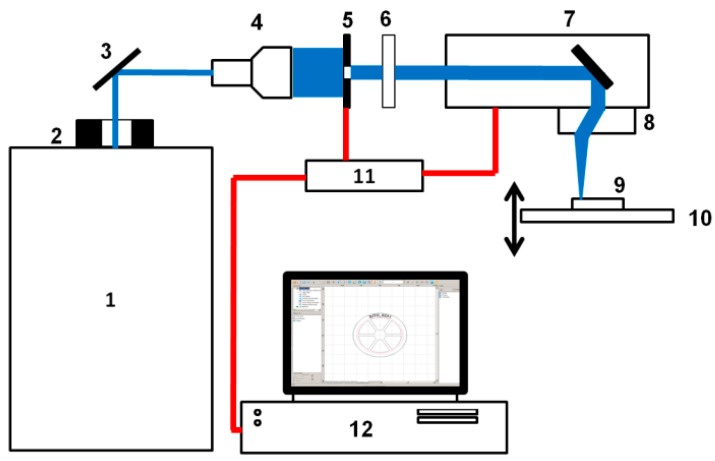
Diagram of a laser system used, which was composed of 1—PL2210/SH/TH/FH picosecond laser (produced by EKSPLA); 2—Thorlabs shutter (Optical Beam Shutter SH05); 3—plane mirror; 4—laser beam polarizing damper; 5—Thorlabs BE05-355 optical beam expander with quintuple magnification; 6—iris diaphragm φ 5.8 mm; 7—SS-IIE10 355 nm galvanometric scanner produced by the company RAYLASE [7]; 8—S4LFT4010/126 SILL telecentric lens; 9—handle; 10—PT3/M Thorlabs sliding table XYZ; 11—RLC USB RAYLASE scanner driver; 12—computer with the weldMARK 3.0 software. The laser beam is marked in blue. Electric signal connections are marked in red.

Graphene paper laser ablation was conducted with the use of a PL2210/SH/TH/FH picosecond laser produced by the company EKSPLA, Vilnius, Lithuania. A diagram of the used laser setup is presented in Figure 1.

The laser beam pulse energy of the first harmonic (wavelength—1064 nm) was 1.3 mJ, on the third one (355 nm) it was 0.45 mJ, and on the fourth one (266 nm) it was 0.25 mJ. The duration of the pulse of the first harmonic was equal to 70 ps, whereas the frequency of repetition was 1 kHz. The energy of the pulses was regulated by the use of a 990-0070-355 polarizing damper produced by the company EKSPLA, Vilnius, Lithuania.

A galvanometric scanner, produced by the company RAYLASE (Weßling, Germany), with a telecentric lens of focal length equal to 100 mm, which focuses radiation, was used on the surface of the processed material. Microsieves were produced using laser beam radiation on the third harmonic (355 nm).

Laser pulse energy and laser pulse numbers of 10, 20, 50 and 100 µJ and 10 to 80 pulses, respectively, during graphene paper laser ablation were selected as independent variables. A hole diameter of ≤10 µm was used as a criterion for a quality assessment of laser ablation. The optimum laser ablation parameter values were determined using the MATLAB program (2017.A, MathWorks, Natick, MA, USA) taking into consideration Equations (1) and (2).

## 3. Results and Discussion

### 3.1. Graphene Paper Testing

**Figure 2:**
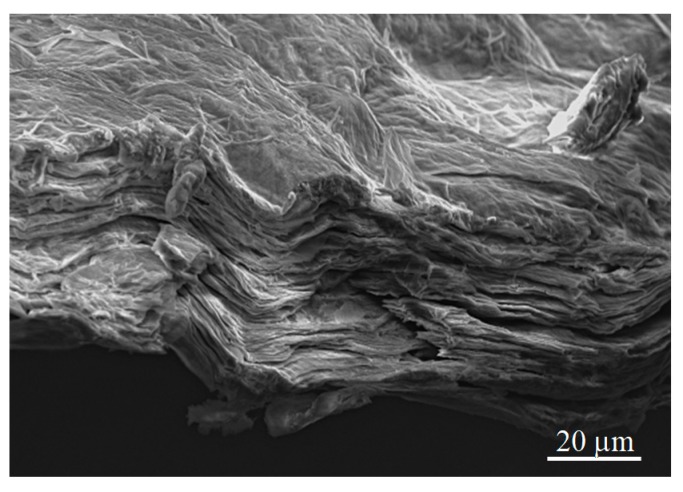
Multilayer structure of graphene paper (3500×).

The morphology of the graphene paper used in these studies is shown in Figure 2. Analysis of the photographs taken with the use of SEM of the specimens showed a visible multilayer structure composed of overlapping graphene layers arranged perpendicularly to the direction of compression.

### 3.2. Selection of Laser Ablation Parameters

**Figure 3:**
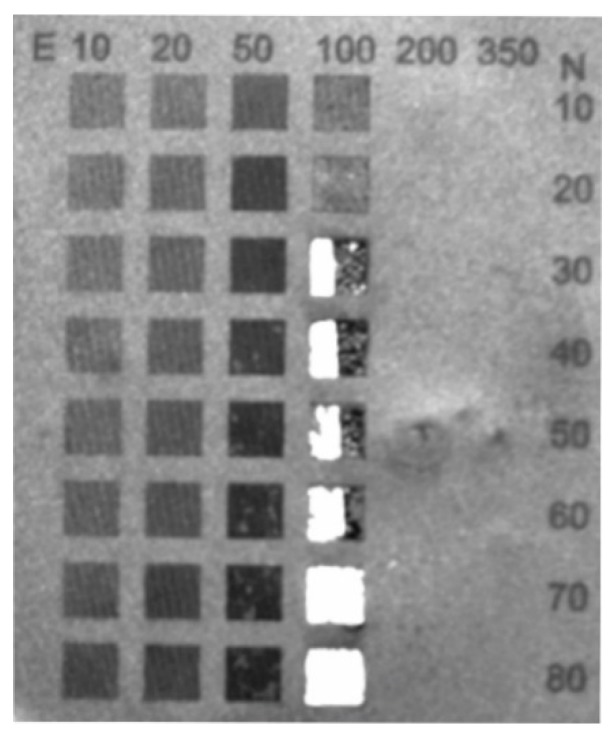
View of the specimens after laser ablation. E—pulse energy in μJ; N—number of laser pulses.

**Figure 4:**
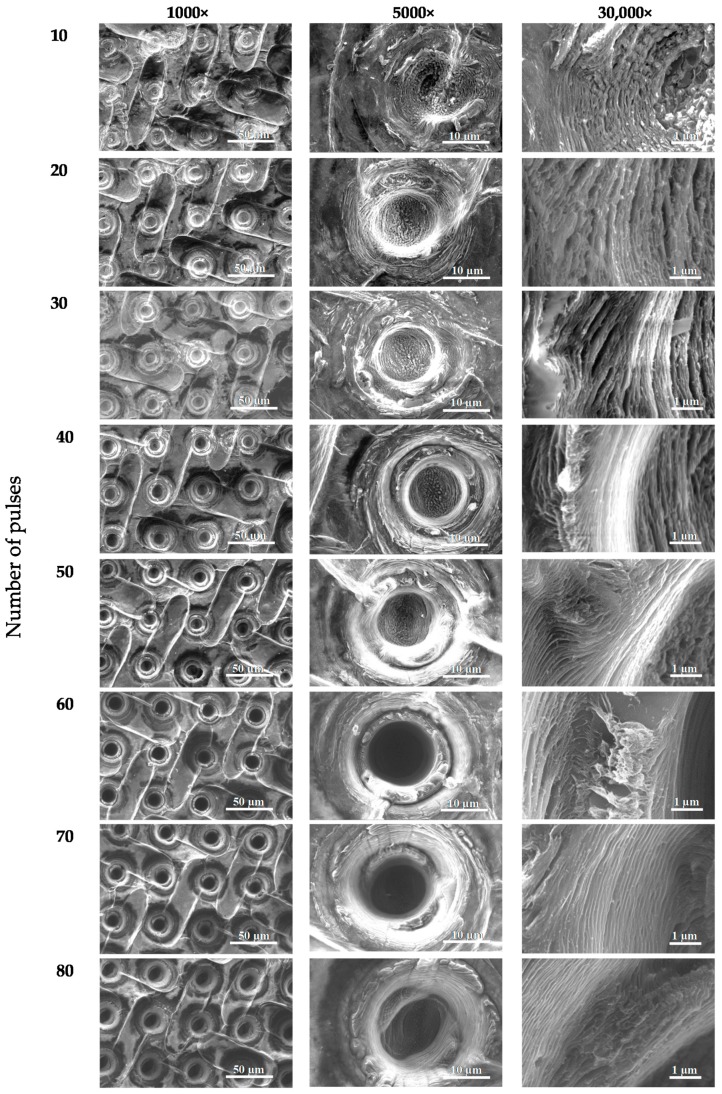
SEM images of the boundary areas of the craters created in graphene paper by a laser ablation method (pulse energy—10 µJ).

**Figure 5:**
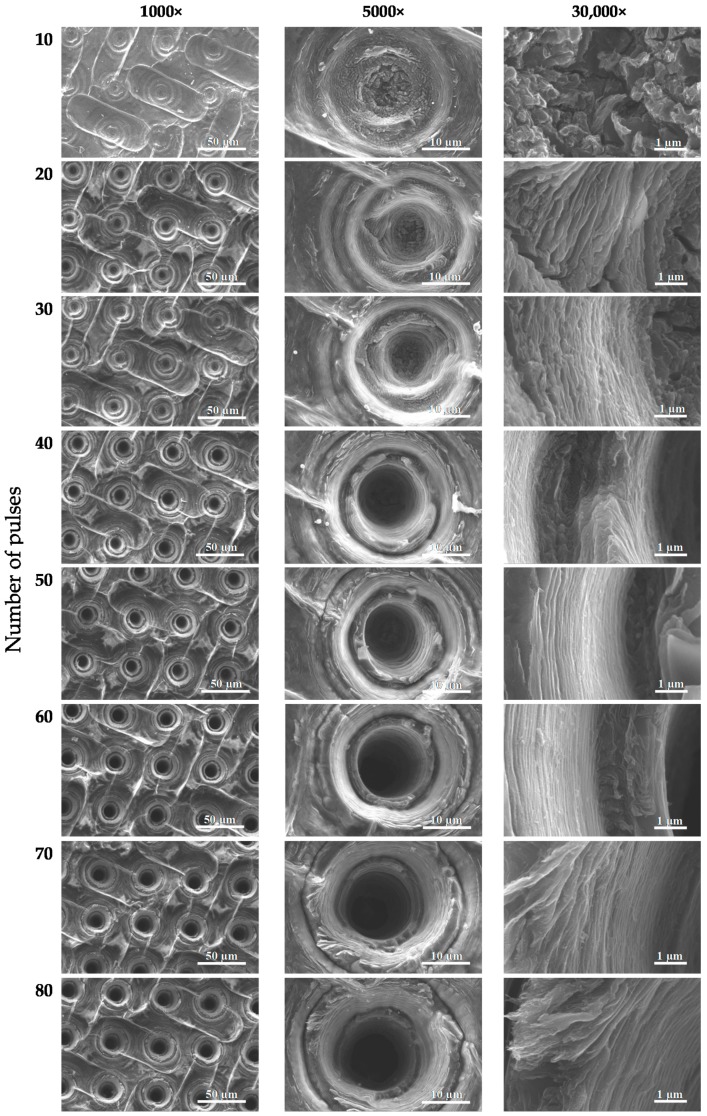
SEM images of the boundary areas of the craters created in graphene paper with a laser ablation method (pulse energy—20 µJ).

**Figure 6:**
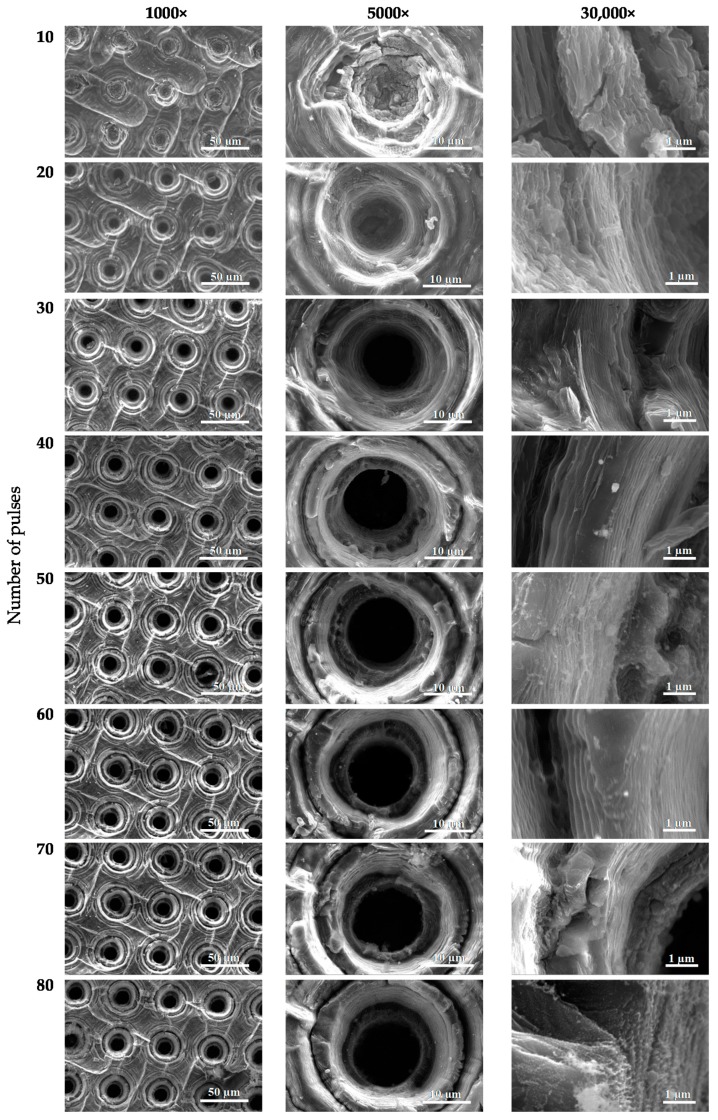
SEM images of the boundary areas of the craters created in graphene paper with a laser ablation method (pulse energy—50 µJ).

During selection of the laser ablation parameters of the graphene paper a degradation of ablated material was observed when laser energy was increased to 100 μJ with a laser pulse number above 30, as is shown in Figure 3. Laser ablation attempts (Figure 3) were next structurally tested and the results of these tests are presented in Figure 4, Figure 5 and Figure 6, while information about hole diameters dependence on laser ablation parameters is provided in Table 1. The microsieves contained up to 100,000 holes with a diameter between 8 and 20 µm.

The results of SEM imaging of the structural changes occurring in graphene paper upon laser ablation are presented in Figure 4, Figure 5 and Figure 6. In the SEM images, there are visible diffraction bands of the laser beam, which affected the tested material. The edges of the holes are characterized by multiple layers of graphene arranged parallel in respect to each other and perpendicularly to the axis of laser beam interaction. SEM images of the graphene paper surface (Figure 4, Figure 5 and Figure 6) revealed a diffraction structure of energy density distribution, which, in the focal plane, closely resemble the Bessel function (Equation (2)).

In Table 1, the diameters of the holes made using different laser pulse energies and a number of pulses are presented. Results presented in Table 1 are shown with one standard deviation calculated from 10 measurements. Diameters below 10 µm are marked in bold.

**Figure 7:**
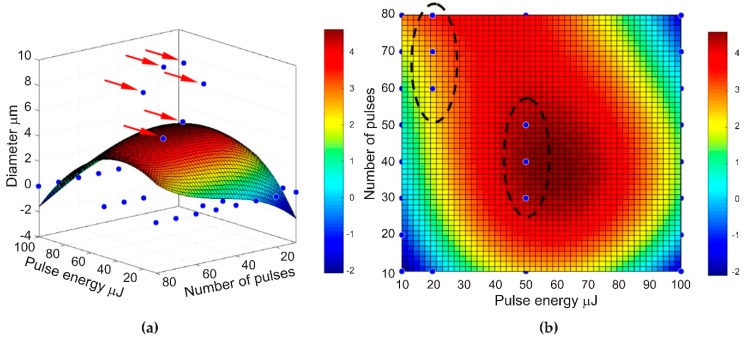
Optimization of graphene paper laser ablation parameters obtained using MATLAB: (**a**) 3D and (**b**) 2D.

In the MATLAB software, the obtained results of the graphene paper laser ablation were used to develop a function determining a range of optimum parameters. For this purpose, value 1 was assigned to sets of holes with diameters in a range of 6–10 µm, whereas value 0 was assigned to the diameters which did not satisfy this condition. Based on the assumptions, a function of the selection of graphene paper laser ablation parameters was developed (Figure 7).

Analysis of the conducted tests proved that holes can be fabricated only at certain parameters of laser processing with diameters in a range of 6–10 µm, which enable the separation of circulating tumor cells. It should be noted that once a hole is created, increasing the number of pulses does not significantly influence any increase in diameter. However, this may cause greater development of the edges (for example, Figure 6; pulse energy—50 µJ, number of shots—80). Laser pulses of high energy, instead of forming holes, caused degradation of graphene paper not only in the exposed area but also around it (as in Figure 3, column 100 µJ, which shows a number of pulses over 20).

### 3.3. Distribution of Energy Density in Laser Beam

Quality improvement in the laser beam was required in the system to achieve a focus as small as possible. For this purpose, an optical beam expander and an iris diaphragm were used. The optical beam expander enlarged the beam five times, whereas the diaphragm cut out its high-quality central part. Distribution of energy density of a pulse passing through the diaphragm was estimated by means of the following equation [24]:

(1) $$\begin{array}{c} e(r)=e_{0}\exp\left[-0.5\cdot {\left(\frac{r}{w_{0}}\right)}^{2}\right]\;,\;r<a,\;r<w_{0},\\ e(r)=0,\;r>a, \end{array}$$

where e_0_ = energy density on the beam axis; a = 2.9 mm—radius of the diaphragm; w_0_ = 2.25 mm—normalization parameter; r = coordinates in the cylindrical system.

The Equation (1) was obtained assuming that a beam incident on the diaphragm has Gaussian distribution of energy density. The cut off Gaussian beam underwent diffraction. The calculations of the distributions were carried out in MATLAB by the Laguerre–Gauss method for the initial distribution described in Equation (1) [24]. The axial symmetry of the beam was assumed.

**Figure 8:**
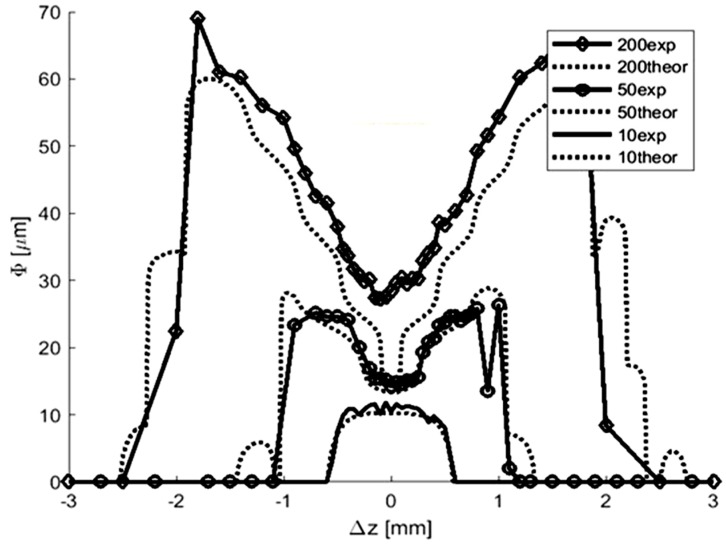
Diameter φ (μm) of the crater drilled in a function of distance Δz (mm) of the exposed surface from the focal plane. Curves with exp marking in the legend concern the experiment, point curves with theor marking concern the calculations. A number at the beginning of the curve name denotes the energy of the pulse (μJ). The laser fluence of each curve is ca. 3.5 J·cm^−2^.

A focused 355 nm laser beam causes microdrilling in graphene paper leading to holes with diameters depending on the pulse energy and distance of the processed surfaces from the focal plane. Measurements of the diameters of the craters engraved by laser pulses of different energies were conducted and calculations of the diameters of those craters, assuming 3.5 J·cm^−2^ as a threshold energy density for engraving, were carried out. The comparison of the experimental and theoretical results is shown in Figure 8.

A good compliance of the results of calculations and measurements occurs at low energies of the pulse. When the energies of the pulse are higher, resulting from interference of thermal conductivity and shock waves, compliance decreases, particularly in the area of the focal plane (curve 200 in Figure 8).

Comparison of the results of the calculations and the experiment (Figure 8) may lead to the conclusion that holes in the focal plane are generated with a laser beam of crosswise distribution of energy density similar to that of Bessel [24]:

(2) $$\begin{array}{c} e(r)=e_{0}{\left[\frac{2J_{1}(x)}{x}\right]}^{2};\;x=\frac{2\pi \;a\;r}{\frac{\lambda }{f}}, \end{array}$$

where *λ* = 355 nm—wavelength of laser radiation; *f* = 100 mm—lens focal length; J_1_ = Bessel’s function of the first kind.

**Figure 9:**
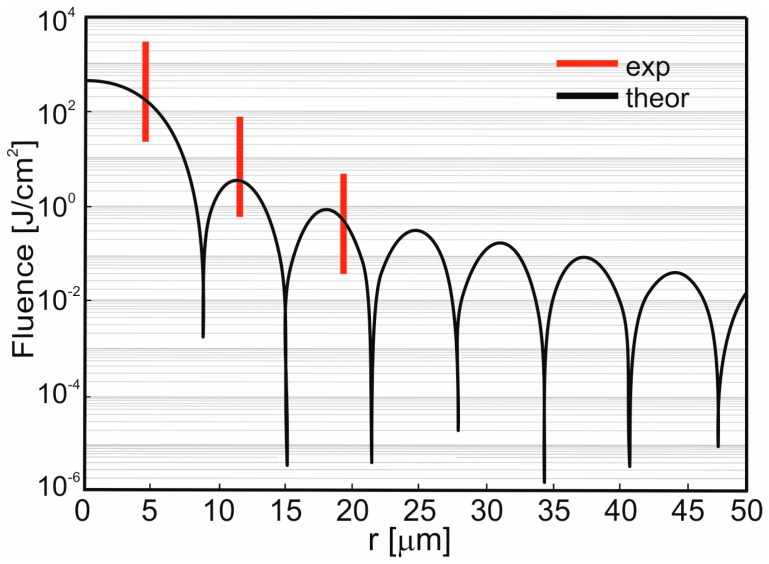
Distribution of laser beam fluence in the focal plane for total pulse energy 200 μJ. The red lines mark the radius of the central hole and position of the maxima of the diffraction rings determined as in Figure 7.

Figure 6 and the Equation (2) demonstrate results where the holes with small diameters may be achieved only by such pulses where energy density in the maximum of the first diffraction ring (r = 5.25 µm in Figure 9) is smaller than the threshold energy density approximately equal to 3.5 J·cm^−2^.

**Figure 10:**
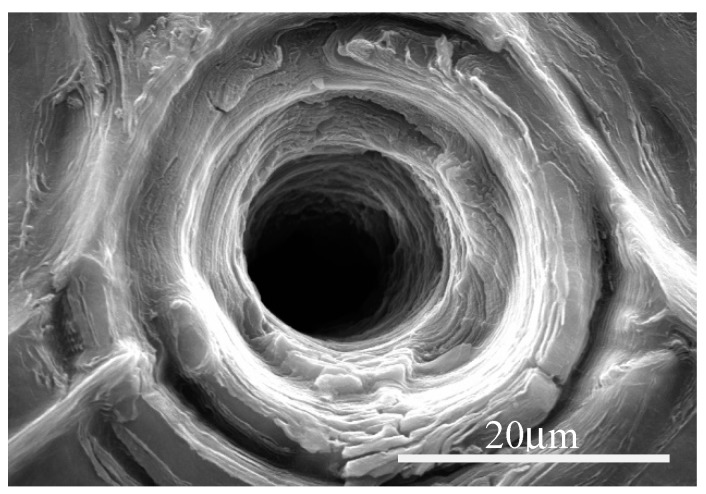
The result of graphene paper microdrilling with the use of a picosecond laser.

Figure 10 presents microscopic photographs of graphene paper in which the holes were made with a laser beam. The paper was placed in the focal plane. Diameters of diffraction rings, visible in Figure 10, are marked in Figure 9.

## 4. Conclusions

1. Graphene paper morphology shown in the image taken on the microscope with the use of a scanning electron microscope (SEM) revealed multi-layered petals of graphene arranged in parallel. Typical lines in Raman spectra, i.e., D, G, D’, D + G, 2D, proved the presence of graphene oxide in the graphene paper;
2. Calculated and measured distributions of the laser beam fluence show a good compliance. The tests conducted on the graphene paper laser ablation process demonstrated that the energy of a laser pulse is a crucial parameter decisive for the result of the processing. The lower the pulse energy, the smaller the diameter of the produced holes and the better the quality of their edges. Increasing the pulse energy decreases the number of the shots required to produce the hole. However, it decreases the quality of the processing and, moreover, causes uncontrolled failure of the processed material due to thermal conductivity and shock wave effects;
3. The results of the test performed also show that after producing the holes, increasing the number of pulses does not significantly affect their diameter any longer;
4. The analyses conducted proved that in order to successfully produce a microsieve from graphene paper, 50 to 70 pulses for one hole must be applied at energy levels of 20 µJ or 30 to 50 pulses at energy levels of 50 µJ.

## Figures and Tables

**Table 1:** The diameters (μm) of the holes for applied graphene paper laser ablation parameters.

**Number of pulses**		**Pulse Energy µJ**			
		**10**	**20**	**50**	**100**
	10	0	0	0	0
	20	0	0	0	0
	30	0	0	**8.1 ± 0.5**	0
	40	0	0	**9.7 ± 0.4**	0
	50	0	**6.8 ± 0.6**	**9.8 ± 0.7**	0
	60	0	**7.1 ± 0.5**	11.3 ± 1.2	0
	70	0	**9.8 ± 0.5**	13.2 ± 1.8	0
	80	0	10.5 ± 1.1	14.5 ± 2.3	0

## Supplementary Materials

The following are available online at https://www.mdpi.com/1996-1944/13/7/1568/s1. Characterization of graphene paper used in the studies by the Laser-Induced Breakdown Spectroscopy (LIBS), Raman Spectroscopy, and Fourier Transform Infrared Spectroscopy (FTIR). The following are available online, Figure S1: LIBS (Laser Induced Breakdown Spectroscopy) spectrum of the graphene paper, Figure S2: Raman spectrum of graphene paper for laser wavelength equal to 532 nm (a) and position of Raman measurement (b), Figure S3: The FTIR spectrum of graphene paper.
