# Supplementary material for: The Influence of Laser Ablation Parameters on the Holes Structure of Laser Manufactured Graphene Paper Microsieves
Source: Materials (Basel). 2020 Mar 28;13(7):1568. doi: 10.3390/ma13071568 (PMC7177287; doi:10.3390/ma13071568)
Supplement: Supplementary file 1 [file materials-13-01568-s001.pdf]

Supplementary Materials

The Influence of Laser Ablation Parameters on the Holes Structure of Laser Manufactured Graphene Paper Microsieves

Barbara Nasiłowska [1,*], Zdzisław Bogdanowicz [2], Antoni Sarzyński [1], Wojciech Skrzeczanowski [1], Małgorzata Djas [3], Bartosz Bartosewicz [1], Bartłomiej J. Jankiewicz [1], Ludwika Lipińska [3], and Zygmunt Mierczyk [1]

[1] Institute of Optoelectronics, Military University of Technology, gen. S. Kaliskiego 2, 00-908 Warsaw, Poland; a.sarzynski9@upcpoczta.pl (A.S.); wojciech.skrzeczanowski@wat.edu.pl (W.S.); bartosz.bartosewicz@wat.edu.pl (B.B.); bartlomiej.jankiewicz@wat.edu.pl (B.J.J.); zygmunt.mierczyk@wat.edu.pl (Z.M.)

[2] Faculty of Mechanical Engineering, Military University of Technology, gen. S. Kaliskiego 2, 00-908 Warsaw, Poland; zdzislaw.bogdanowicz@wat.edu.pl

[3] Łukasiewicz Research Network–Institute of Electronic Materials Technology, Department of Chemical Synthesis and Flake Graphene; Wólczyńska 133, 01-919 Warsaw, Poland; malgorzata.djas@itme.edu.pl (M.D.); ludwika.lipinska@itme.edu.pl, (L.L.)

* Correspondence: barbara.nasilowska@wat.edu.pl

Received: 6 March 2020; Accepted: 27 March 2020; Published: 28 March 2020

Characterization of graphene paper used in the studies

Laser-Induced Breakdown Spectroscopy

The chemical composition of graphene paper used in these studies was determined using the LIBS (Laser-Induced Breakdown Spectroscopy) method [1]. A laser beam (Quantel Brio Nd:YAG laser) of 80 mJ energy with a wavelength equal to 1064 nm was focused on the test specimen of the material causing its ablation and, subsequently, heating and ionization of the generated vapours and plasma generation. Radiation emitted by the plasma was recorded by an optical fiber with the use of the ESA 4000 spectrometer with an iCCD (intensified charge-coupled-device) camera mounted in its focal plane.

The LIBS spectrum of graphene paper specimen, obtained by the accumulation of three laser shots, is presented in Figure S1. We observed dominating atomic and molecular carbon transitions and weak Mg, Al, Si, and Ca lines in the spectrum, which may correspond to impurities left from the production process or can be related to the conditions of the graphene paper storage. Clearly visible CN bands were also present in LIBS spectra of graphene compounds reported in [2,3]. High molecular CN bands shown in Figure S1, between 380 and 388 nm, are very similar or almost identical to those presented in [3].

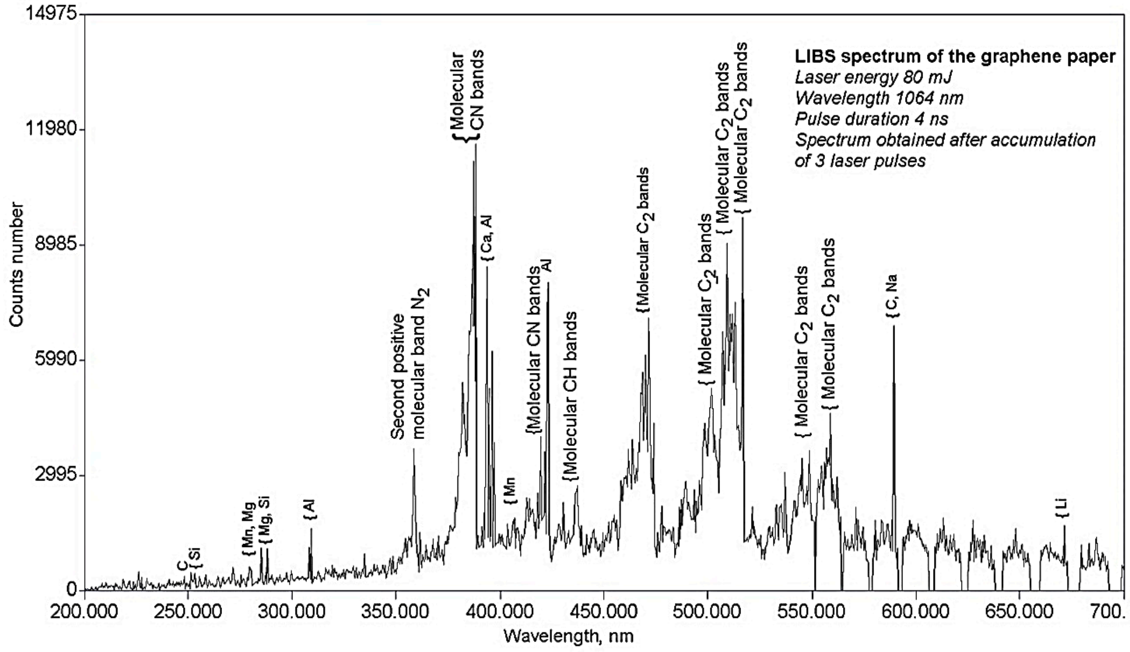

Figure S1. LIBS (Laser-Induced Breakdown Spectroscopy) spectrum of the graphene paper.

Raman spectroscopy

The Raman spectra were acquired by using a Renishaw InVia Raman microscope equipped with an Andor EMCCD detector. The Raman signal was collected using laser radiation with a wavelength of 532 nm and laser excitation power of 2.5 mW on the sample. The laser beam was directed to the sample through a 20× objective lens and the laser spot size was ca. 5 μm in a diameter. The wavelength of the instrument was calibrated using an internal silicon wafer, and the spectrum was centered at 520.5 cm^{-1}.

In the Raman spectrum of the graphene paper, a typical spectral pattern was observed. Basic bands were observed: D, G, and weak D′, 2D, as well as D + G [4]. In these cases, the Raman shift for a laser wavelength of 532 nm was 1360 cm^{-1}, 1600cm^{-1}, 1760cm^{-1}, 2680 cm^{-1}, and 2940 cm^{-1}, respectively (Figure S2).

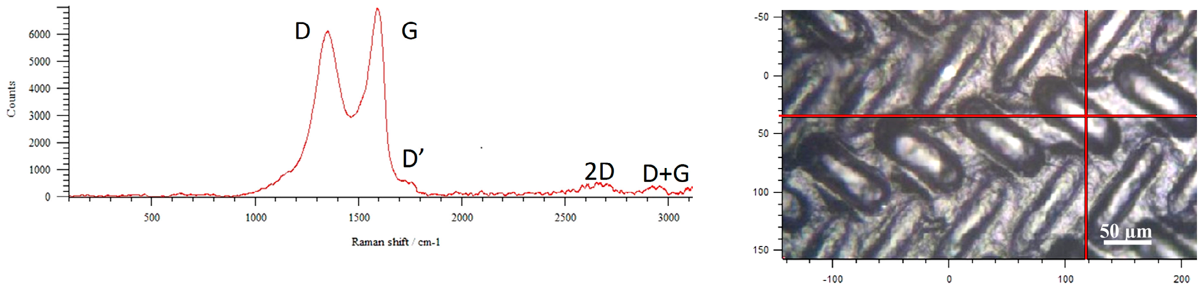

(**a**) (**b**)

Figure S2. Raman spectrum of graphene paper for laser wavelength equal to 532 nm (**a**) and position of Raman measurement (**b**).

Diffuse Reflectance Infrared Fourier Transform Spectroscopy (DRIFTS)

Diffuse Reflectance Infrared Fourier Transform Spectroscopy (DRIFTS) technique of the FTIR method, which provides a convenient and rapid method of sample preparation, was applied [5,6]. DRIFTS spectra were obtained using a Perkin-Elmer Spectrum GX Optica FTIR spectrometer. The

measurement range was 4000–650 cm^{-1} (2.5–12.5 μm) with a 4 cm^{-1} resolution. The authors chose the non-destructive DRIFTS technique to avoid destruction of the graphene paper structure.

Significant peaks were found in the DRIFTS spectrum of graphene paper (Figure S3). Characteristic peaks corresponding to the absorption due to bands of graphene oxide were confirmed at 3473 cm^{-1} (O–H stretching vibrations), at 1756 cm^{-1} (stretching vibrations from C=O), at 1661 cm^{-1} and 755 cm^{-1} (skeletal vibrations from C=C aromatic rings), at 1468 cm^{-1} (C–H stretching vibrations), at 1303 cm^{-1} and 1124 cm^{-1} (C–C stretching vibrations), and 861 cm^{-1} (C–H out of plane deformations).

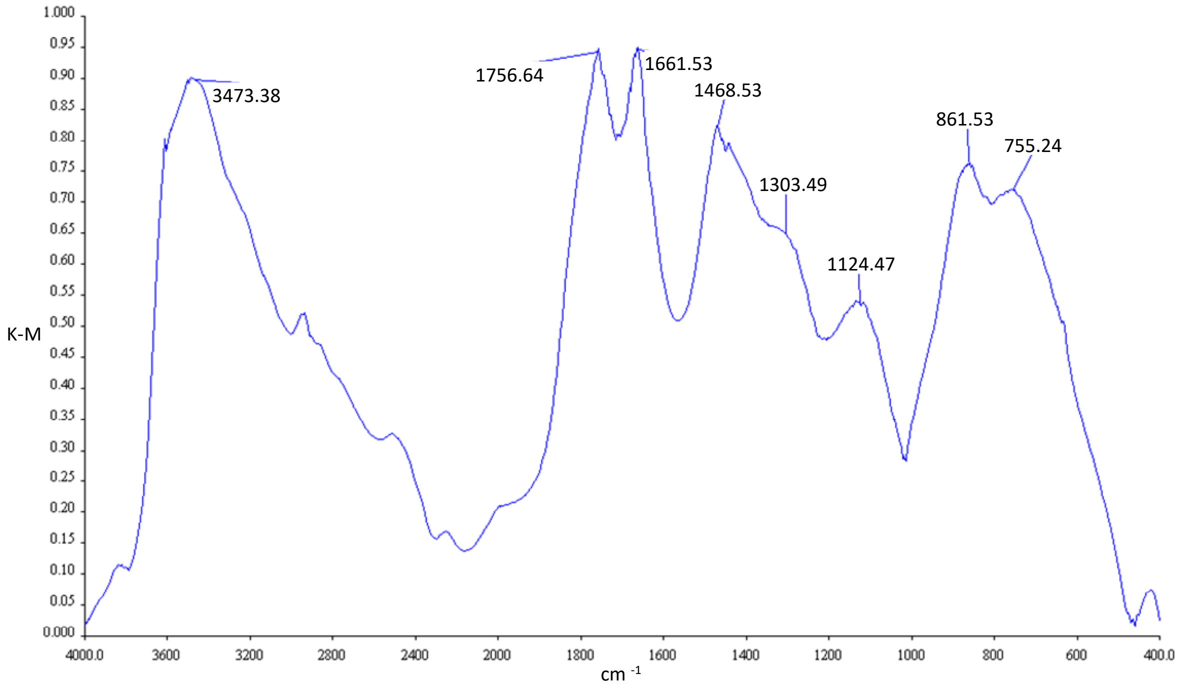

Figure S3. The Diffuse Reflectance Infrared Fourier Transform Spectroscopy (DRIFTS) spectrum of graphene paper.

References

1. Miziolek, A.; Palleschi, V.; Schechter, I. *Laser-Induced Breakdown Spectroscopy*; Cambridge University Press: Cambridge, UK, 2006.
2. Lebedev, V.F.; Rabchinskii, M.K.; Kozlyakov, M.S.; Stepanov, D.N.; Shvidchenko, A.V.; Nikonorov, N.V.; Vul, A. Laser-induced breakdown spectroscopy: an advanced method for analysis of nanocarbon materials chemical composition. *J. Anal. At. Spectrom.* **2018**, *33*, 240–250.
3. Serrano, J.; Cabalín, L.M.; Moros, J.; Laserna, J.J. Potential of laser-induced breakdown spectroscopy for discrimination of nano-sized carbon materials. Insights on the optical characterization of graphene. *Spectrochim Acta B*. **2014**, *97*, 105–112.
4. Backes, C. et al. Production and processing of graphene and related materials. *2D Mater.* **2020**, *7*, 022001.
5. Carraher, C.E. Physical characterization of polymeric materials. In *Applied Polymer Science: 21st Century*; Craver, C.D., Carraher, C.E., Eds.; Elsevier Inc.: Amsterdam, the Netherlands, 2000.
6. Mularczyk-Oliwa, M.; Bombalska, A.; Kaliszewski, M.; Włodarski, M.; Kopczyński, K.; Kwaśny, M.; Szpakowska, M.; Trafny, E.A. Comparison of fluorescence spectroscopy and FTIR in differentiation of plant pollens, *Spectrochimica Acta Part A*. **2012**, *97*, 246–254.
